# Lysozyme improves gut performance and protects against enterotoxigenic *Escherichia coli* infection in neonatal piglets

**DOI:** 10.1186/s13567-018-0511-4

**Published:** 2018-02-20

**Authors:** Guangping Huang, Xiangqing Li, Dan Lu, Shen Liu, Xun Suo, Qiuyan Li, Ning Li

**Affiliations:** 1 State Key Laboratory of Agrobiotechnology & College of Veterinary Medicine, China Agricultural University, Beijing, China; 2 State Key Laboratory of Agrobiotechnology & College of Biological Sciences, China Agricultural University, Beijing, China; 3 Shenzhen Sunsmile Biotechnology Co., Ltd, Shenzhen, Guangdong China; 4 Shanghai Institute of Medical Genetics, Shanghai Children’s Hospital, Shanghai, China; 5 School of Life Science and Engineering, Foshan University, Foshan, Guangdong China

## Abstract

Diarrhea remains one of the leading causes of morbidity and mortality globally, with enterotoxigenic *Escherichia coli* (ETEC) constituting a major causative pathogen. The development of alternative treatments for diarrhea that do not involve chemotherapeutic drugs or result in antibiotic resistance is critical. Considering that lysozyme is a naturally occurring antimicrobial peptide, in a previous study we developed a transgenic pig line that expresses recombinant human lysozyme (hLZ) in its milk. In the present study, we examined the protective effects of the consumption of this milk against ETEC infection in neonatal piglets. We found that consuming hLZ milk facilitated faster recovery from infection and decreased mortality and morbidity following an ETEC oral inoculation or infection acquired by contact-exposure. The protective effect of hLZ was associated with the enrichment of intestinal bacteria that improve gut health, such as *Lactobacillus*, and the enhancement of the mucosal IgA response to the ETEC-induced diarrhea. Our study revealed potential protective mechanisms underlying the antimicrobial activity of human lysozyme, validating the use of lysozyme as an effective preventive measure for diarrhea.

## Introduction

Enterotoxigenic *Escherichia coli* (ETEC) is one of the most devastating pathogens associated with diarrhea in animals and humans, annually accounting for 157 000 deaths among children [1]. ETEC pathogenicity is dependent on colonizing factors (also known as adhesins) and enterotoxins [2]. ETEC that is associated with diarrhea in neonatal pigs expresses one or more fimbrial adhesins, including F4 (K88), F5 (K99), F6 (987P), F7 (F41) and F18, while ETEC in older pigs most commonly produces F4 (K88) or F18 fimbriae. Fimbrial adhesins bind to glycoprotein receptors on the enterocyte brush border, enabling *E. coli* colonization in the small intestine. The bacteria then secrete heat-labile enterotoxins and/or heat-stable enterotoxins that alter tight junction integrity and disrupt the paracellular passages of ions, solutes and water, leading to diarrhea [3, 4]. Persistent diarrhea results in dehydration and malnutrition, and may increase the chance of subsequent infection by other pathogens. ETEC shed in the feces can survive for at least 5–6 months [5, 6], affording opportunities to contaminate other animals, plants, or water.

As widespread resistance is emerging for many antibiotics, the development of alternative diarrhea treatments that do not involve chemotherapeutic drugs or result in antibiotic resistance is crucial. Increasing studies have focused on the use of natural anti-bacterial proteins, such as lysozyme, lactoferrin and secretory IgA, which can be found in the tears, saliva, and milk of all mammals, and which provide non-specific antimicrobial and immune-stimulating activities [7–10]. As one of the main host defense factors, lysozyme directly kills bacteria by hydrolyzing the glycosidic β-(1–4) linkage between *N*-acetylmuramic acid and *N*-acetylglucosamine of the peptidoglycan polymer in the cell wall [11–13]. In addition, lysozyme can also regulate immune function by directly or indirectly modulating the complement system, and can enhance the function and proliferation of polymorphonuclear neutrophils and phagocytes [14, 15]. These effects suggest that lysozyme can be used to protect hosts against infectious diseases.

Although large volumes of cow and goat milk are available for human consumption, both are low in certain key health-promoting antimicrobial components such as lysozyme. Human milk contains a 1500- to 4000-fold more lysozyme (400 mg/L) than cow milk (0.13 mg/L) or goat milk (0.25 mg/L) [16]. The lysozyme concentration in pig milk is even lower, at less than 0.065 mg/L [17]. Advancements in gene transfer technologies have made it possible to breed transgenic cows and goats that express recombinant human lysozyme (hLZ) in their milk, incorporating this protective protein into a readily available product that can be used by both people and animals [10, 18–20].

We recently generated a line of transgenic pigs that express high levels of recombinant human lysozyme in their milk, with an average concentration of 1300.7 ± 126.7 mg/L (range from 1140.5 to 1576.8 mg/L) [21, 22]. To test whether the milk confers protective effects, neonatal piglets were assigned to wild type sows or to sows that expressed human lysozyme in their milk. Half of the piglets were deliberately challenged with ETEC, and the other half was permitted to acquire the infection passively from their challenged littermates. Piglets were then monitored for diarrhea and other signs of pathology.

## Materials and methods

### Bacterial strain and culture

Enterotoxigenic *Escherichia coli* K88 (O149:K91, K88ac) was purchased from the China Veterinary Culture Collection Center (CVCC, Beijing, China). The virotype profile is O149:F4:LT:STb:Stx2e:eae [23]. Cultures were grown in Luria–Bertani (LB) medium containing 1% tryptone, 0.5% yeast extract and 1% NaCl, and incubated at 37 °C with shaking for 16 h. Cultures were harvested by centrifugation at 3000 × *g* for 10 min, washed 3 times with sterile phosphate-buffered saline (PBS, pH = 7.2), re-suspended in sterile PBS, and serially diluted to approximately 2 × 10^8^ CFU/mL for oral challenge as described previously [24].

*Micrococcus lysodeikticus* and *Staphylococcus aureus* were purchased from the China General Microbiological Culture Collection Center (CGMCC, Beijing, China) and cultured in LB medium at 28 and 37 °C, respectively, in shake flasks.

To measure the bactericidal effects of lysozyme, 2.5 mL of *E. coli* cell suspension with an optical density (OD_600_) of approximately 0.7 was incubated with 100 μL of defatted milk from hLZ-transgenic or wild type pigs. The inhibitory effect of lysozyme on the growth of Gram-positive and Gram-negative bacteria was tested using *S. aureus* and *E. coli*, with *M. lysodeikticus* as a positive control. OD_600_ was recorded at 1, 3, 5, and 7 h after incubation and compared with the value at 0 h.

### Experimental design

Five hLZ transgenic and 5 wild-type sows of the same parity were used in the experiment. All sows were confirmed seronegative for K88 antibodies using a commercial enzyme-linked immunosorbent assay (ELISA) kit, following the manufacturer’s instructions (Shanghai Yaji Biological Technology Co., Ltd., Shanghai, China), as described previously [24]. Sixty wild-type female piglets with similar birth weights (1.62 ± 0.15 kg) were randomly assigned to one of the ten sows (six piglets per sow) at 1 day of age. The environment was maintained at 30 ± 2 °C and all piglets were allowed to nurse. During the 17-day lactation period, sow milk was the only source of nutrition for the piglets. All piglets had free access to water.

For each sow, three of the six piglets were orally challenged at 3 days of age with a dose of 5 mL PBS containing approximately 1 × 10^9^ CFU of ETEC O149: K88, delivered via a stomach tube connected to a 5 mL syringe. These three piglets are hereafter described as “challenged”. The dose of ETEC used in this study causes diarrhea within 6 h in young piglets (data not shown). After receiving the ETEC, the three piglets were returned to their pen in order to contact-expose their three littermates, referred to throughout the manuscript as cohabited piglets. The beginning of the experiment is defined at this point (time = 0). In summary, the piglets were assigned to four experimental groups of 15 piglets each, defined by two milk sources (hLZ transgenic milk [hLZ] vs. wild-type milk feeding [WT]), and two infection states (ETEC challenged vs. cohabited).

After 7 days, the challenged piglets were euthanized, and the three cohabited piglets remained with their assigned sow. Piglets were weighed on days 0, 3, 5, 7, and 14.

### Clinical scoring

Disease signs were scored as described in a previous study scored as described in a previous study [25]. Piglets were monitored daily by a pathologist blinded to the treatments. A disease score of 0 indicated no signs of diarrhea, lethargy or dehydration; 1, feces softer than normal, but with overall demeanor unaffected; 2, feces liquid with little or no lethargy or dehydration; 3, appearance of prolonged diarrhea, with obvious dehydration and/or lethargy; and 4, diarrhea with severe, life-threatening dehydration and lethargy. Piglets were considered dehydrated if a pinch of skin at the base of the neck did not return to its original shape. Piglets were identified as lethargic if they were motionless in the pen or required stimulation to move. Piglets were considered to have diarrhea when the disease score was at a level of 2 or greater, and piglets with a disease score of 4 for at least 2 consecutive days were recorded as deaths and humanely euthanized with an intraperitoneal injection of pentobarbitone to minimize suffering [25], as they were unlikely to recover. Rectal temperature was registered daily from the challenge day onwards, and measurements were taken prior to any other manipulation. The incidence of diarrhea (%) was calculated as a percentage of the number of newly affected piglets during the experimental period divided by the total number of piglets in each group [25].

### Detection of fecal ETEC K88 from challenged piglets by polymerase chain reaction (PCR)

Fecal samples from challenged piglets were examined for ETEC K88 6 h after the challenge. Bacterial DNA was extracted using a QIAmp DNA stool kit (Qiagen, Valencia, CA, USA), following the manufacturer’s instructions. Adhesin gene F4 was detected by PCR using forward primer 5′-GCTGCATCTGCTGCATCTGGTATGG-3′ and reverse primer 5′-CCACTGAGTGCTGGTAGTTACAGCC-3′, as described previously [26].

### Histological examination of the intestinal morphology

All cohabitated piglets were euthanized on day 14 of the experiment. The small intestine was divided into three segments: duodenum, to about 5–8 cm from the pylorus; jejunum, the middle portion; and ileum, the distal section approximately 5 cm in length proximal to the ileocecal junction. After being gently rinsed with ice-cold PBS (pH = 7.2), the segments were immediately fixed in 4% paraformaldehyde, embedded in paraffin, sliced into 4 μm thick sections, and stained with hematoxylin and eosin. Villus length and crypt depth were measured using Image-Pro Plus software (Image-Pro Plus 6.0; Media Cybernetics, Silver Spring, MD, USA). Ten well-oriented villi were selected and measured in triplicate for each piglet.

### Analysis of the distribution and expression of tight junction proteins by real-time PCR

The tight junction (TJ) is formed by a protein complex and is widely used as a marker of intestinal integrity [27]. Mucosal tissues from the duodenum, jejunum and ileum were scraped with a glass slide, immediately placed in liquid nitrogen, and stored at −80 °C. Real-time PCR analysis for TJ mRNA expression was performed using a SYBR Premix Ex Taq II qPCR kit (TaKaRa Biotechnology, Dalian, China) on a LightCycler480 thermocycler (Roche, Mannheim, Germany). Gene-specific primers for the amplicons of zonula occludens (ZO)-1, occludin, and β-actin are listed in Table 1. Relative transcript levels were quantified by the 2^−∆∆CT^ method as described previously [28].

**Table 1:** **Primers used for real-time PCR analysis**

Gene	Primers sequences	Size in base pairs
ZO-1	F: 5′-TGAGTTTGATAGTGGCGTTG-3′	298
	R: 5′-TGGGAGGATGCTGTTGTC-3′	
Occludin	F: 5′-CTAGTCGGGTTCGTTTCC-3′	167
	R: 5′-GACTGATTGCCTAGAGTGT-3′	
β-actin	F: 5′-TGCGGGACATCAAGGAGAAGC-3′	273
	R: 5′-ACAGCACCGTGTTGGCGTAGAG-3′	

### Analysis of gut microbiota by 16S rRNA sequencing

Fresh fecal samples were obtained on days 0, 3, 5, 7, and 14 from the cohabitated piglets in each feeding group. The collected material was mixed well to produce a single homogeneous sample, and then divided into 3 equal parts. All fecal samples were stored at −80 °C prior to bacterial DNA extraction. The V3 hypervariable region of the 16S rRNA gene was amplified by PCR using a forward primer (5′-GATCCTACGGGAGGCAGCA-3′) and reverse primer (5′-GCTTACCGCGGCTGCTGGC-3′) [29]. Sequencing libraries were generated using the NEBNext^®^ Ultra™ DNA Library Prep Kit (NEB, USA), following the manufacturer’s recommendations. The library was sequenced on an Illumina MiSeq platform [30].

To analyze gut microbial diversity, operational taxonomic units (OTUs) were clustered with a 97% similarity cutoff using UPARSE (version 7.1). The taxonomy of each 16S rRNA gene sequence was assigned using the Ribosomal Database Project Classifier against the Silva (SSU128) 16S rRNA database with a confidence threshold of 70% [31]. Metastats was used to confirm differences in taxonomic abundance between the two groups.

### Quantitation of IgA and pro-inflammatory cytokines by ELISA

Fecal extracts collected on days 7 and 14 were concentrated fourfold by freeze-drying and resuspending with sterile water. Secretory IgA (sIgA) antibody to *E. coli* K88ac was quantified using indirect ELISA assay kits from Shanghai Yaji Biological Technology Co., Ltd., Shanghai, China. Serum was separated from clotted blood on days 0, 7, and 14 and preserved at −20 °C until use. IL-6 and TNF-α were quantitated using commercially available ELISA kits (Shanghai Yaji Biological Technology Co., Ltd., Shanghai, China). All ELISA procedures were performed as recommended by the manufacturer’s instructions. Each sample was assayed in triplicate.

### White blood cell (WBC) counts

White blood cell subpopulations (including lymphocytes, monocytes, and neutrophils) were quantified with a Hemavet hematology analyzer (Drew Scientific, Dallas, Texas, USA).

### Statistical analysis

All experimental data were analyzed using SPSS software (V. 19.0; SPSS Inc., Chicago, IL, USA). Data were expressed as mean ± SEM. Statistical significance was determined using two-tailed Student’s unpaired *t* test. Differences were considered significant at *P* values less than 0.05.

## Results

### Reduced severity and duration of diarrhea in ETEC K88 challenged and cohabitated piglets fed with hLZ milk

#### Challenged infection

**Figure 1:**
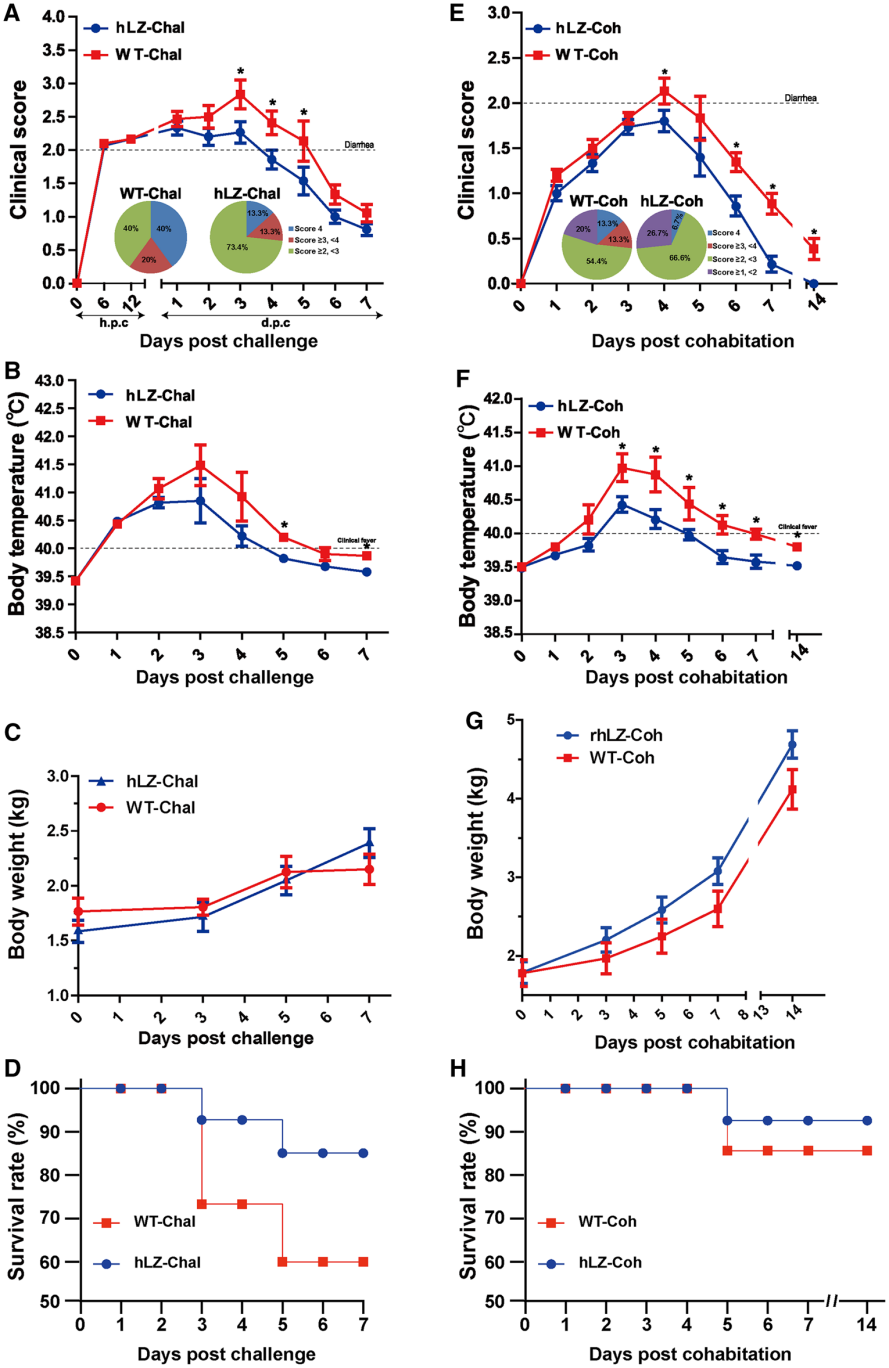
**Piglets fed with hLZ milk were protected from ETEC.** The left column **A**–**D** represents data collected from ETEC challenged piglets, and the right column **E**–**H** represents cohabited piglets that passively acquired ETEC from challenged littermates. Time zero is defined as the point at which piglets were challenged and penned with the unchallenged (cohabited) piglets. Clinical scores for challenged (**A**) or cohabitated (**E**) piglets. Rectal temperature was monitored daily (**B**, **F**). The body weight gains were measured at the indicated times (**C**, **G**). Survival rates (**D**, **H**). Note that all challenged piglets were euthanized on day 7. Asterisk indicates *P* < 0.05. hLZ: hLZ milk-fed piglets, WT: control milk-fed piglets, Chal: challenged, Coh: cohabited.

All piglets in WT milk feeding group and hLZ milk-feeding group exhibited diarrhea (score ≥ 2) within 6 h after the challenge with ETEC K88 (Figure 1A). PCR analysis indicated that all challenged pigs excreted ETEC in their feces (data not shown). Compared to the hLZ-fed group, piglets in the WT-fed group showed more severe and extensive diarrhea, especially by days 3 and 5 (*P* < 0.05, Figure 1A). Twelve out of 15 piglets (80%) belonging to the WT-fed group displayed high fever (> 40 °C, *P* < 0.05) and growth retardation (Figures 1B and C). Six piglets (40%) displayed severe diarrhea (score = 4) with life-threatening dehydration and apparent lethargy, and were considered as deaths and euthanized on days 3 and 5 (Figure 1D). However, most of the piglets (73.4%, 11/15) that consumed hLZ transgenic milk suffered from mild diarrhea (score = 2), and their symptoms abated 3 days later. Only 2 piglets (13.3%, score = 4) failed to recover from the diarrhea and were euthanized on days 3 and 5 (score = 4, Figure 1D). Notably, pigs treated with the hLZ milk appeared to resume growth on day 5 (Figure 1C). All challenged piglets were euthanized on day 7.

#### Cohabitation infection

The cohabitated (i.e., unchallenged) piglets were contact-exposed to the challenged piglets from the onset of the experiment. Within 7 days, all cohabited piglets in both feeding groups showed subnormal body weight gain and increased body temperature (Figures 1E–G). Diarrhea (score ≥ 2) occurred in 12 out of 15 (80%) cohabitating piglets fed with WT milk, and it persisted for at least 2 days longer (*P* < 0.05, Figure 1E), along with a higher body temperature (*P* < 0.05, Figure 1F) and poor growth (Figure 1G), compared with the hLZ-fed group. Diarrheal symptoms became aggravated in 2 piglets, resulting in death on day 5 (13.3%; score = 4, Figure 1H). In contrast, the hLZ-fed group displayed transient diarrhea and most diarrheal piglets were apparently healthy after exposure to ETEC-contaminated feces. Only one piglet (6.7%) developed severe diarrhea (score = 4, Figure 1H).

Overall, piglets in the hLZ-fed group experienced diarrhea for a shorter time, with lower clinical scores and incidence than the piglets in the WT-fed group, suggesting that the consumption of human lysozyme improved the outcome.

### Lysozyme protects against ETEC-induced intestinal mucosal damage

**Figure 2:**
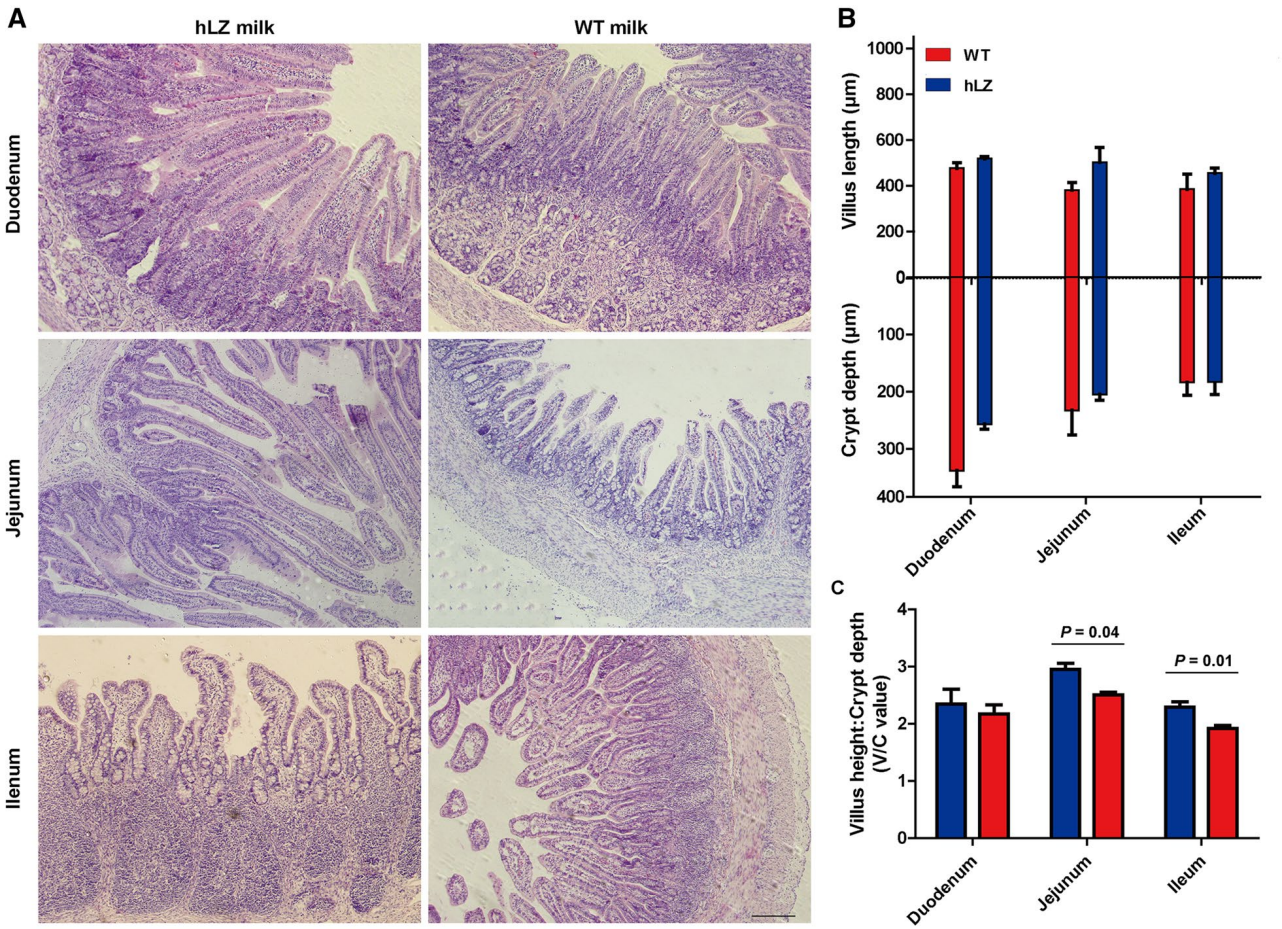
**hLZ alleviates ETEC-induced intestinal damage. A** Representative histological sections of the intestinal segments from cohabited piglets fed hLZ milk or wild type milk. Tissues were collected on day 14. Scale-bar = 200 µm. Villus length and crypt depth (**B**) and the ratios of villus length to crypt depth (**C**) at the duodenum, jejunum and ileum from the piglets fed with hLZ or WT milk are shown as mean ± SEM (*n* = 10).

The morphology of three intestinal sections from the cohabitated piglets was examined at the end of the experiment (day 14; Figure 2A). Milk source effects were assessed using measurements of villus length, crypt depth, and the ratio (villus length/crypt depth) of these values (Figures 2B and C). In the jejunum and ileum, the ratio was significantly higher in hLZ fed than in WT fed piglets. However, the ratio in the duodenum was statistically indistinguishable between the two feeding groups, although it is slightly higher in hLZ fed animals. We conclude that the WT milk-fed piglets display obvious pathological damage, including microvillus loss and eosinophil infiltration (Figure 2A), in comparison with the hLZ fed group.

**Figure 3:**
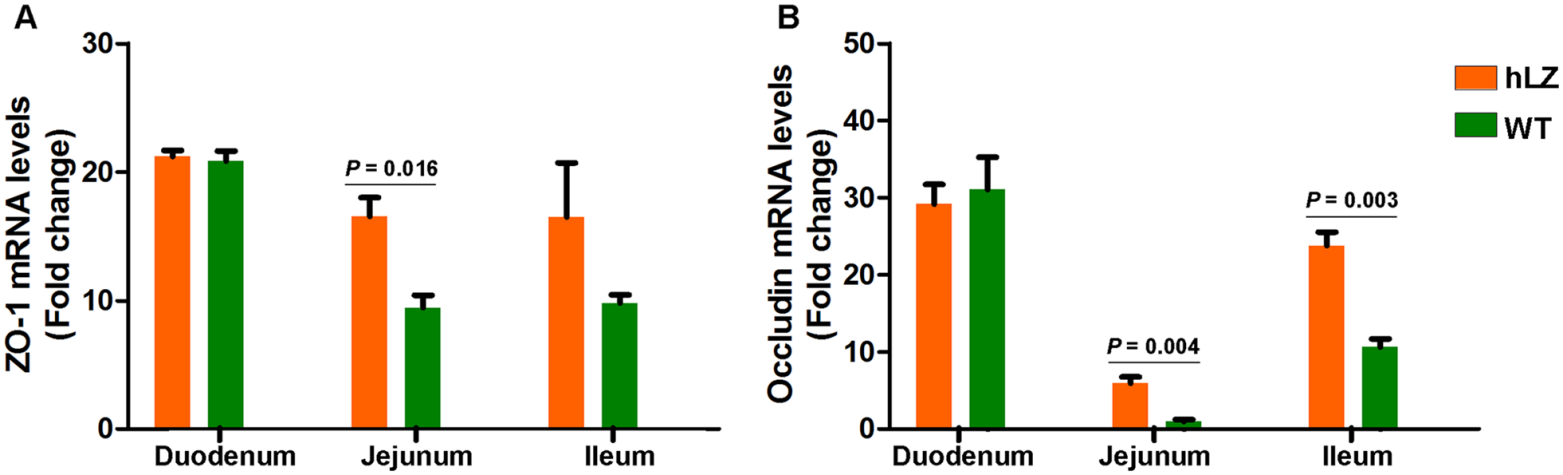
**Tight junction proteins ZO-1 (A) and Occludin (B) mRNA levels in the intestinal mucosa (including duodenum, jejunum, and ileum) of cohabited piglets determined by real-time PCR.** Data are shown as mean values ± SEM. *P* < 0.05 was considered statistically significant. hLZ: hLZ milk-fed piglets, WT: control milk-fed piglets.

To further evaluate the integrity of the intestinal apical membrane, the expression of ZO-1 and occludin were measured at the end of the experiment using quantitative real-time PCR. Compared to hLZ milk-fed piglets, ZO-1 and occludin were significantly depleted in the WT-fed group, especially in the jejunum and ileum (Figure 3), indicating that hLZ protects piglets from ETEC K88-induced severe intestinal mucosal injury.

Together, these results suggest that hLZ milk positively affects intestinal morphology without compromising the integrity of the TJ in the apical membrane.

### Protection against ETEC infection does not depend on the lytic function of lysozyme

**Figure 4:**
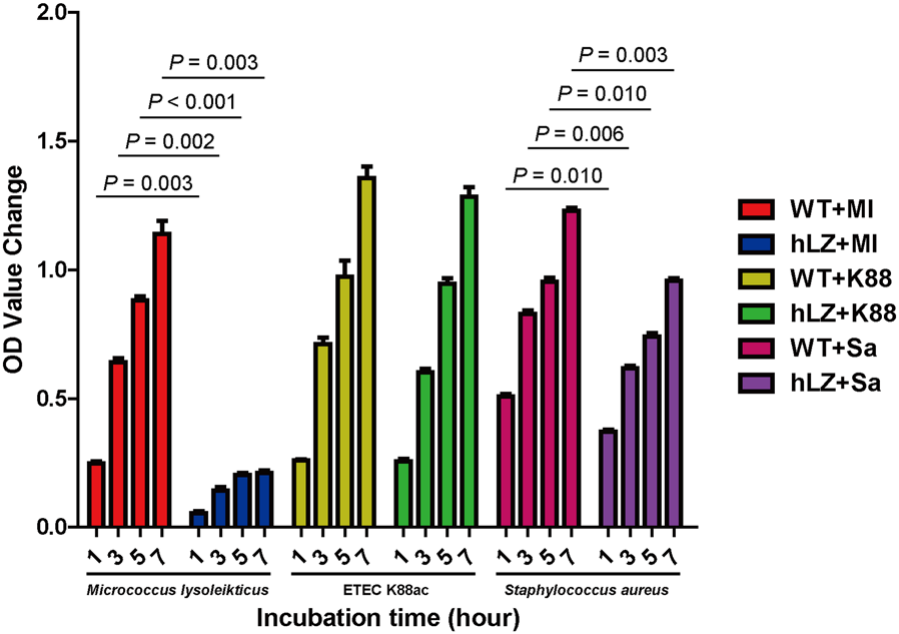
**hLZ inhibits the growth of Gram-positive bacteria (*Micrococcus lysodeikticus*** **and** ***Staphylococcus aureus*) with less influence on Gram-negative bacteria (*Escherichia coli*** **K88).** Bacteria were cultured in Luria–Bertani medium for 8 h, diluted to approximately 2000 CFU/mL, and incubated with 100 μL of defatted milk from transgenic pigs. Controls were incubated with the same volume of defatted WT milk. OD_600_ was recorded after 1, 3, 5, and 7 h and then compared with the value at 0 h. WT + Ml: *Micrococcus lysodeikticus* treated with WT milk; hLZ + Ml: *Micrococcus lysodeikticus* treated with hLZ transgenic milk; WT + K88: *Escherichia coli* K88 incubated with WT milk; hLZ + K88: *Escherichia coli* K88 incubated with hLZ milk; WT + Sa: *Staphylococcus* *aureus* incubated with WT milk; hLZ + Sa: *Staphylococcus* *aureus* incubated with hLZ milk. *P* < 0.05 was considered statistically significant.

Cell wall destruction by lysozyme in Gram-negative bacteria is less severe than Gram-positive bacteria due to the protective effects of outer membrane lipopolysaccharides. Although the concentration of lysozyme in the transgenic milk is more than 1000 mg/L, the inhibitory effects on the growth of K88 in vitro were negligible (Figure 4). In contrast, the growth of the Gram-positive species *M. lysodeikticus* (*P* < 0.05) and *S. aureus* (*P* < 0.05) was significantly inhibited. *M. lysodeikticus* was considerably more sensitive to lysozyme since the cell walls are composed of a peptidoglycan polymer.

### Significant enrichment of probiotics in the gut is associated with lysozyme consumption

**Figure 5:**
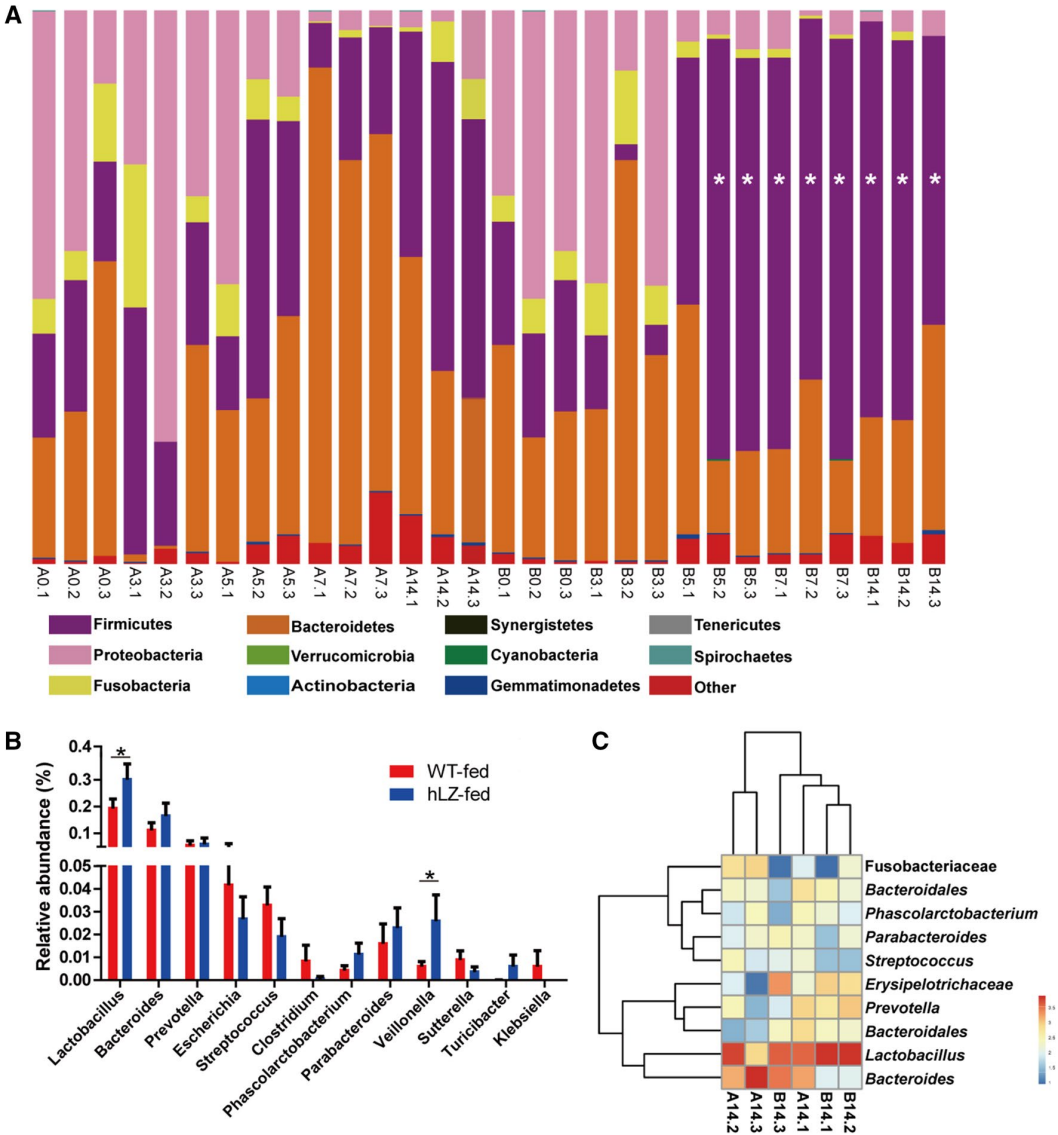
**Consumption of hLZ milk increases the abundance of probiotics (*Lactobacillus*) in the intestine of cohabited piglets. A** Relative phylum abundance of gut microbiota from WT and hLZ milk-fed piglets on days 0, 3, 5, 7, and 14. **B** Relative abundance of bacterial genera in the guts of WT and hLZ milk-fed piglets at day 14. **C** Taxonomic classifications at the genus level for the fecal samples collected at day 14. Only the 10 most enriched classes are shown. **A** The fecal samples collected from WT milk-fed piglets at days 0 (A0), 3 (A3), 5 (A5) 7 (A7) and 14 (A14); **B** the fecal samples collected from hLZ milk-fed piglets at days 0 (B0), 3 (B3), 5 (B5) 7 (B7) and 14 (B14). The samples from each day were divided into three equal parts and measured as replicates. Asterisk indicates *P* < 0.05.

To examine the effects of hLZ milk on intestinal flora, changes in the intestinal microbiome were monitored using 16S rRNA gene sequencing. Fecal microbial profile showed that all piglets started with similar fecal microbial populations, but the profiles changed significantly after 5 days of hLZ milk consumption (Figure 5A). Animals receiving hLZ milk had an underrepresentation of Bacteroidetes were underrepresented and Firmicutes were overrepresented in the animals receiving hLZ milk (Figure 5A). Within the Firmicutes, the genus *Lactobacillus* in hLZ-fed pigs was present in a significantly greater proportion and was apparently higher than in WT-fed piglets on both days 7 and 14 (*P* < 0.05, Figures 5B and C). Conversely, three genera (*Clostridium*, *Streptococcus*, and *Escherichia*) that are associated with disease apparently increased over time in WT-fed pigs (Figure 5B). The results demonstrated that hLZ milk modulated intestinal microbiota by increasing the ratio of beneficial bacteria and decreasing the numbers of disease-causing microbe.

### An enhanced mucosal immune response plays a crucial role in the protection against ETEC infection

**Figure 6:**
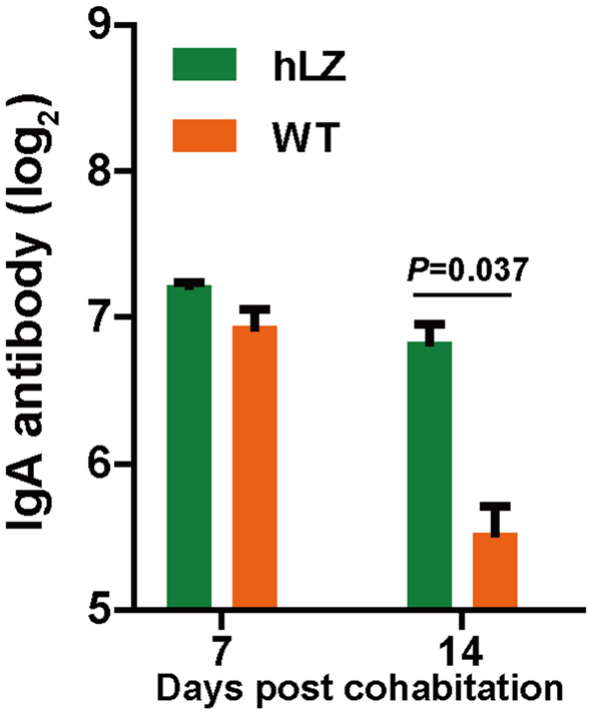
**hLZ enhances mucosal IgA immune response to ETEC K88 infection.** hLZ: hLZ milk-fed piglets, WT: WT milk-fed piglets.

sIgA is an indicator of mucosal immunology. Fecal sIgA in the WT- and hLZ-fed groups was assayed on days 7 and 14. We found that the sIgA levels in hLZ milk-fed piglets were slightly higher on day 7 and increased significantly on day 14 (*P* = 0.037) relative to WT-fed control (Figure 6), indicating that the mucosal immune response was enhanced following the consumption of hLZ milk.

### Decreased inflammatory response to ETEC K88 infection in hLZ-fed pigs

**Figure 7:**
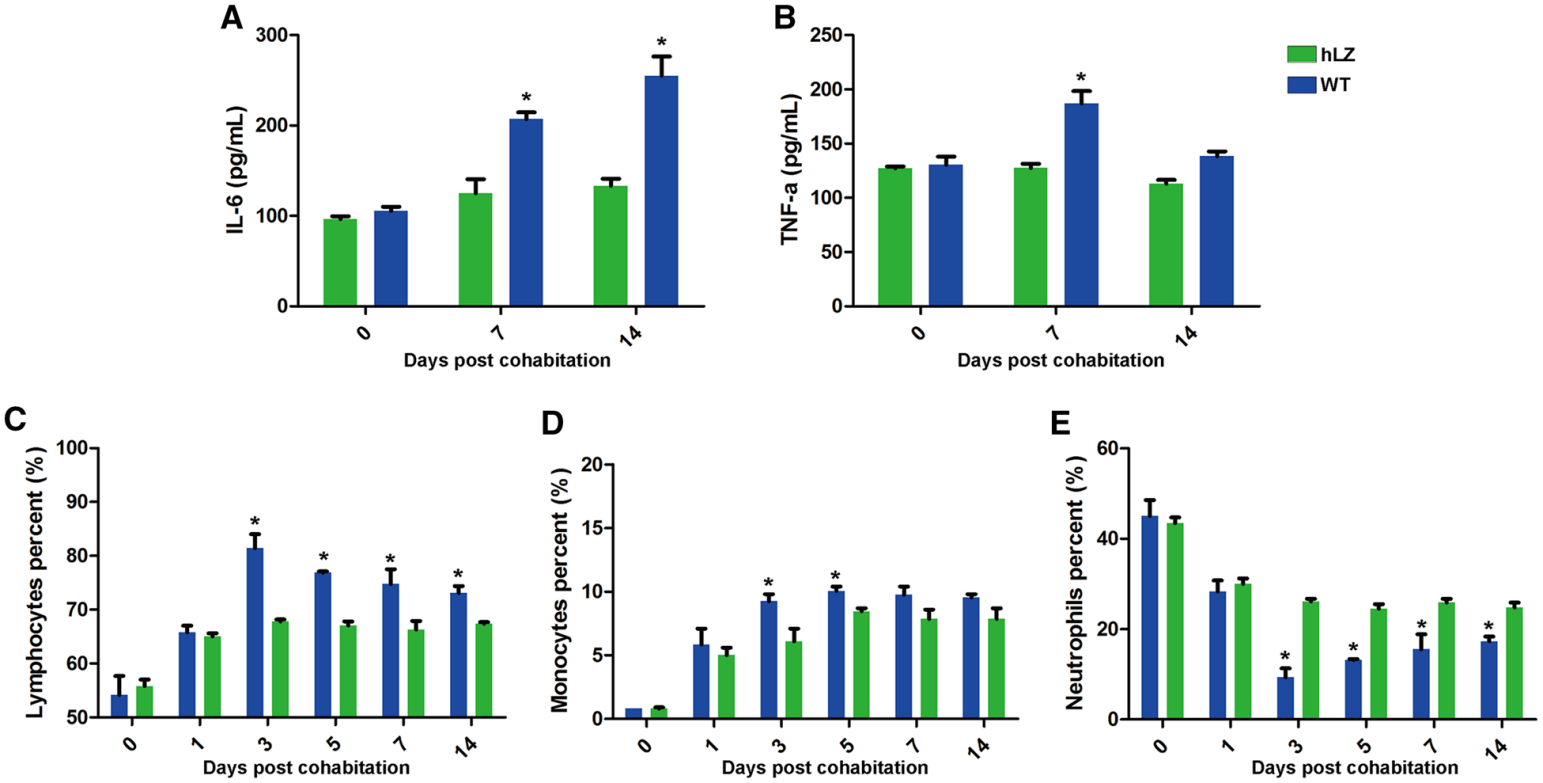
**hLZ decreases inflammatory response to ETEC infection.** The serum levels of pro-inflammatory cytokines IL-6 (**A**) and TNF-α (**B**) secreted by cohabiting pigs was determined by ELISA. Concentrations of lymphocytes (**C**), monocytes (**D**) and neutrophils (**E**) in the cohabiting pigs over the course of the study were determined by WBC analysis. Each sample was assayed in triplicate. Values are mean ± SEM. Asterisk indicates *P* < 0.05.

The secretion of pro-inflammatory cytokines IL-6 and TNF-α in cohabiting piglets increased after exposure to ETEC-contaminated feces. However, piglets fed hLZ-milk showed significantly lower serum levels of both IL-6 (*P* < 0.05) and TNF-α (*P* < 0.05) 7 and 14 days after exposure to ETEC, indicating a limited inflammatory response to ETEC infection (Figures 7A and B).

WBC count analysis showed that hLZ-milk fed piglets tended to have significantly more neutrophils (*P* < 0.05) and fewer lymphocytes and monocytes than those of WT-fed piglets from day 3 after exposure to ETEC (Figures 7C–E).

## Discussion

We examined the ability of lysozyme, a natural antimicrobial protein, to protect neonatal piglets against ETEC K88 infection. We found that the consumption of hLZ milk helped ETEC-challenged piglets to recover from infection faster with lower mortality, and helped cohabitated piglets to decrease the morbidity with less intestinal damage, and enhanced IgA production and *Lactobacillus* enrichment. These findings demonstrate that the introduction of human lysozyme played a beneficial role in gut health and improved the ability of the piglets to resist bacterial infections.

Although lysozyme is well known for its robust antimicrobial activity against Gram-positive bacteria, and to a much lesser degree against Gram-negative bacteria, its bactericidal effect on ETEC K88 in vitro is modest due to the presence of lipopolysaccharides on the outer membrane of *E. coli* K88 (Figure 4) [31–33]. The protective effects observed in vivo against ETEC K88 infection could therefore be due to the regulatory function of lysozyme in the intestinal environment.

Interestingly, nonpathogenic bacteria often encode Ivy, PgdA, Oat, and Pat *O*-acetyltransferases, which are involved in regulating lysozyme resistance [34]. Consistent with a report that found that the consumption of lysozyme-rich milk contributes to the development of intestinal bacteria associated with gut health [19], we found an increase in the abundance of *Lactobacillus* in hLZ-fed piglets. *Lactobacillus* has been shown to improve the intestinal environment and activate intestinal mucosal immunity in many species, resulting in enhanced IgA production [35, 36], which is required for the prevention of fimbriae-mediated colonization and the maintenance of intestinal barrier function. In contrast, ETEC infection in the WT-feeding group increased the levels of *Clostridium*, *Streptococcus*, and *Escherichia*, typically characterized as opportunistic pathogenic bacteria [37]. Lysozyme is also important for limiting systemic inflammation, resulting in decreased inflammatory-driven pathology [34, 38, 39], which is consistent with the lower production of pro-inflammatory cytokines in the hLZ-fed pigs in our study.

ETEC is a significant cause of diarrhea either in neonatal or early-weaned piglets, resulting in severe economic losses in the swine industry [40]. Although several vaccines have been successfully developed for weaned pigs, diarrhea always occurs early after weaning, often within 3–10 days [41]. It would be ideal to vaccinate during the sucking period. However, apart from the ability to resist gastrointestinal degradation, another challenge for ETEC vaccines is to overcome neutralization by maternal antibodies and/or other milk factors [41].

The use of antibiotics at sub-therapeutic levels throughout the swine industry improves performance and overall health. However, increasing concerns regarding antibiotic resistance have made their use problematic [42], and research into alternatives is therefore essential. Lysozyme, as a natural antimicrobial treatment, has been extensively used as a feed additive to increase growth and feed efficiency [43]. As shown in our study, the consumption of lysozyme by neonatal pigs enriches intestinal commensal bacteria and enhances the mucosal IgA response that reinforces the mucus barrier to prevent the penetration of ETEC K88 into epithelial cell surfaces. Furthermore, the high level of neutrophils contributes to the clearance of ETEC, leading to decreased pro-inflammatory cytokine production and limited *E. coli*-induced inflammation. Our findings indicate that lysozyme may be a viable alternative to traditional sub-therapeutic antibiotic use in swine production. To date, multiple lines of transgenic dairy animals that produce lysozyme have been developed to improve animal welfare and growth performance [10, 18, 20–22, 44, 45]. Recent advances in transgenic technologies have removed many of the technical barriers to the predictable and efficient genetic engineering of agricultural species, although there are many political and regulatory hurdles to overcome before genetic engineering can be used in agriculture [46, 47].

In conclusion, the data in this study demonstrate that human lysozyme is beneficial for the gut performance of neonatal piglets and improves resistance to bacterial infections, providing an effective preventive measure for diarrhea.
